# HPLC, NMR and MALDI-TOF MS Analysis of Condensed Tannins from *Lithocarpus glaber* Leaves with Potent Free Radical Scavenging Activity

**DOI:** 10.3390/molecules13122986

**Published:** 2008-12-04

**Authors:** Liang Liang Zhang, Yi Ming Lin

**Affiliations:** 1 Key Lab of Ministry of Education for Coast and Wetland Ecosystems, Xiamen 361005, P.R. China; E-mails: zhll20086@gmail.com (L-L. Z.); linym@xmu.edu.cn (Y-M. L.); 2 Department of Biology, School of Life Sciences, Xiamen University, Xiamen 361005, P.R. China

**Keywords:** *Lithocarpus glaber*, Condensed tannins, Thiolysis, MALDI-TOF, Free radical scavenging activity

## Abstract

Using acid-catalyzed degradation in the presence of cysteamine, the condensed tannins from *Lithocarpus glaber* leaves were characterized, following thiolysis, by means of reversed-phase HPLC, ^13^C-NMR and matrix-assisted laser desorption/ionization time of flight mass spectrometry (MALDI-TOF MS) analyses. The thiolysis reaction products showed the presence of the procyanidin (PC) and prodelphinidin (PD) structures. The ^13^C-NMR spectrum revealed that the condensed tannins were comprised of PD (72.4%) and PC (27.6%), and with a greater content of *cis* configuration rather than the *trans* configuration of C2–C3. The MALDI-TOF MS analysis proved the presence of PD units, and the maximum degree of polymerization (DP) was an undecamer. The antioxidant activity of condensed tannins from *L*. *glaber* leaves was evaluated by using a free radical scavenging activity assay.

## Introduction

**Figure 1:**
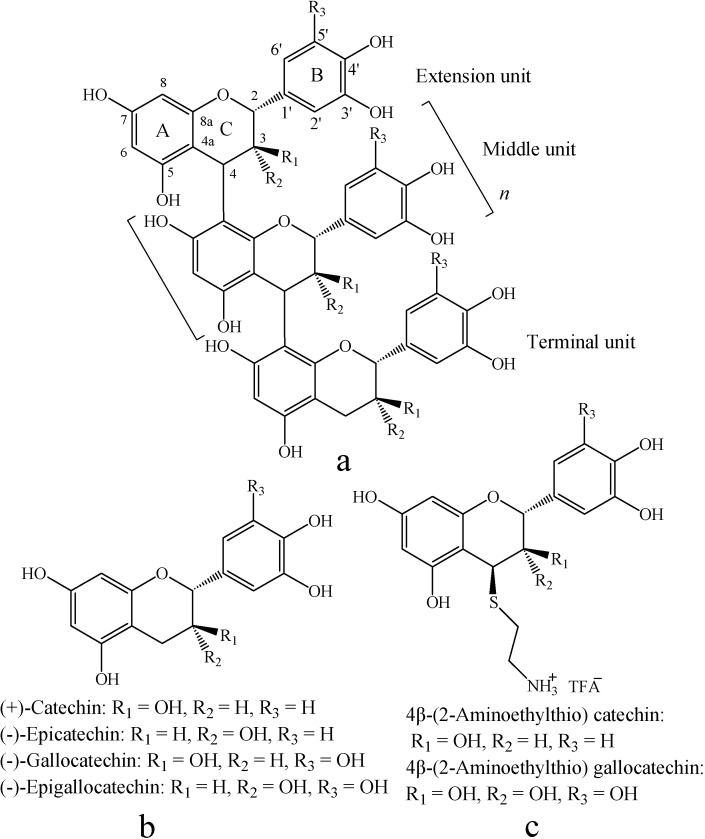
Structures of proanthocyanidins polymer (a), monomeric flavan-3-ol (b), and their aminomethylthio derivatives (c).

Condensed tannins, as an important class of secondary metabolites, are distributed ubiquitously in both gymnosperms and angiosperms [1]. Condensed tannins are polyphenolic natural products composed of ﬂavan-3-ol sub-units linked mainly through C4–C8 (or C4–C6) bonds [2] (Figure 1). The structural diversity of condensed tannins is due to the different sub-units, interﬂavonoid bond position, branching and the presence of non-ﬂavonoid substituents such as gallic acid and sugars [3]. In addition, condensed tannins also vary markedly in molar mass distribution. The extractable condensed tannin polymers in the plants may be composed of molecular species with a wide range of molar masses up to 20,000 (about 40 ﬂavan-3-ol units) [4]. The bioactivity capacity of condensed tannins is generally recognized to be largely dependent on their structure and particularly the degree of polymerization [5].

Tannins have received considerable attention in the fields of nutrition, health and medicine, largely due to their physiological activity, such as antioxidant activity [6], anti-microbial effects [7], and anti-inflammatory properties [8]. Tannins are antioxidants often characterized by reducing power [9] and free radical scavenging activities [10]. The antioxidant capabilities of tannins depend on: (1) the extent of their colloidal state, (2) the ease of interflavonoid bond cleavage or its stereochemical structure, (3) the ease of pyran ring (C-ring) opening, and (4) the relative numbers of –OH groups on A and B rings [11]. Compounds with a trihydroxyl structure in the B-ring display the greatest antioxidant activity [12].

*Lithocarpus glaber* (Fagaceae) is one of the dominant species in the subtropical evergreen broadleaf forests of China. A previous study showed that some Fagaceae species exhibited high tannin levels [13]. Fagaceae tannins consist primarily of condensed tannins or proanthocyanidins [14], but their chemical, biological and pharmacological properties have not yet been determined. In this study, the condensed tannins from *L*. *glaber* leaves were characterized by using acid-catalyzed degradation, ^13^C- NMR and MALDI-TOF MS analysis. In addition, the potent free radical scavenging activity was evaluated and compared with both natural and synthetic antioxidants.

## Results and Discussion

### Total phenolics and condensed tannins contents

*L*. *glaber* leaves had high tannin levels. The total phenolics and condensed tannins contents were 545.21 ± 17.53 mg/g and 401.06 ± 5.04 mg/g, respectively. Polyphenols are the major plant compounds with antioxidant activity. The results of this study strongly suggest that phenolics are important components of this plant, and some of its pharmacological effects could be attributed to the presence of these valuable constituents.

### Thiolysis with cysteamine followed by reversed-phase HPLC

**Figure 2:**
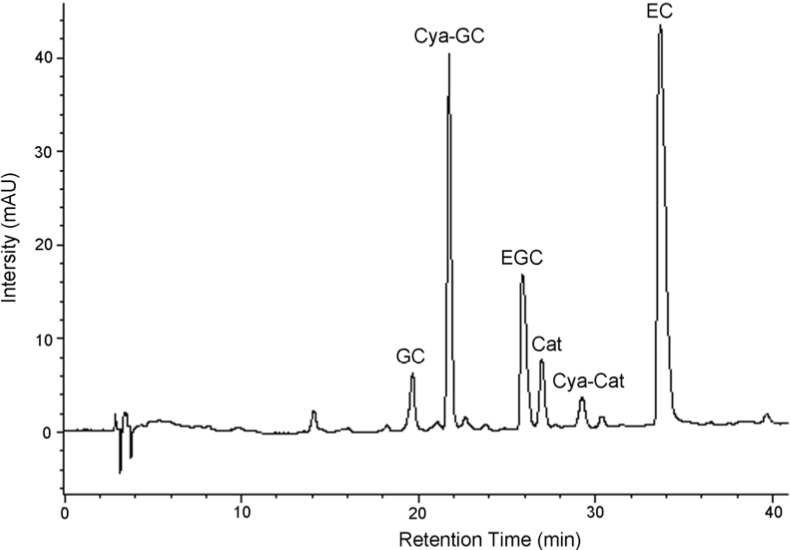
Reversed-phase HPLC chromatograms of condensed tannins from *L*. *glaber* leaves degraded in the presence of cysteamine; EC, (-)-epicatechin; Cat, (+)-catechin; EGC, (-)-epigallocatechin; GC, (-)-gallocatechin; Cya-Cat, 4β-(2-aminoethylthio)catechin; Cya-GC, 4β-(2-aminoethylthio) gallocatechin.

The constituent flavanoid units in condensed tannins from *L*. *glaber* leaves were identified by degradation of these compounds using acid hydrolysis in the presence of cysteamine. Degradation of these compounds with acids in the presence of various nucleophiles is a well known method since the stereochemistry at C2 and C3 positions is preserved [15]. Although acid-catalyzed thiolysis has been used by many researchers [16,17,18], the procedure is lengthy and has an offensive odor. However, the use of cysteamine as the nucleophile is more convenient and offers a better separation of the degradation products when using different chromatographic systems [19]. It takes 45 min to complete the hydrolysis of condensed tannins from *L*. *glaber* leaves. During acid depolymerization in the presence of cysteamine hydrochloride, the interflavan bonds are protonated and broken, leaving the terminal unit intact and the extension unit (including middle unit) as a carbocation [20]. The carbocation is then captured either α or β to the C-ring, producing a monomer aminomethylthio derivative. The depolymerization products were then separated on reversed-phase HPLC (Figure 2).

The flavan-3-ol and their cysteamine conjugates discussed below were identified by the comparison of their retention times, and in some cases UV spectra, with those of authentic standards. The condensed tannins of *L*. *glaber* leaves contained prodelphinidin as well as procyanidin. They also contained the highest proportion of gallocatechin extension subunits. Analysis of the condensed tannins degradation products by reversed-phase HPLC showed that (+)-catechin, (-)-epicatechin, (-)-gallocatechin, (-)-epigallocatechin, 4β-(2-aminoethylthio)catechin, and 4β-(2-aminoethylthio)-gallocatechin were present (Table 1, Figure 2). The extension and terminal units in condensed tannins of *L*. *glaber* leaves, therefore, are (+)-catechin, (-)-gallocatechin, (-)-epicatechin, and (-)-epigallocatechin.

**Table 1:** Concentration of terminal and extension units of condensed tannins of *L*. *glaber* leaves determined by HPLC following thiolysis degradation.

	Concentration (mg/g dried tannins)
Aminoethylthio catechin	8.38 ± 0.58
Catechin	18.53 ± 0.63
Epicatechin	222.73 ± 2.51
Aminoethylthio gallocatechin	258.72 ± 4.21
Gallocatechin	65.93 ± 2.22
Epigallocatechin	247.08 ± 7.15

Values are means ± SD, n = 3.

### ^13^C-NMR analysis of condensed tannins

^13^C-NMR spectroscopy has been employed to examine the nature of polymeric condensed tannins [4]. The ^13^C-NMR spectra of condensed tannins present information on the absolute and relative stereochemistry of the heterocyclic ring (C2–C3), the structures of the chain terminating ﬂavan-3-ol units, the ratio of procyanidin (PC) to prodelphidin (PD) extension units, and the number average molar mass (Mn), according to Czochanska *et al*. [21].

**Figure 3:**
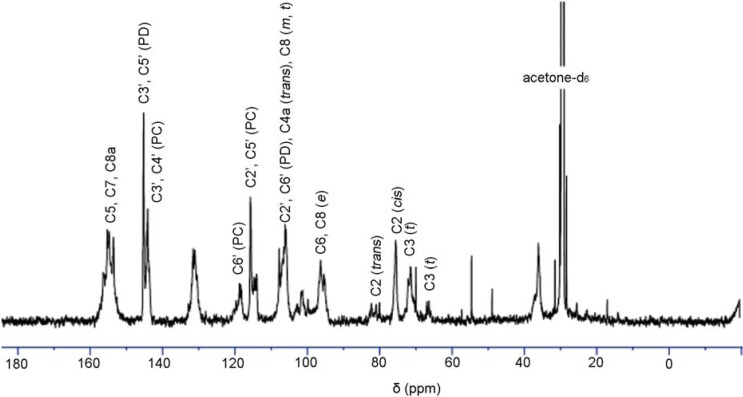
^13^C-NMR (150 MHz) spectrum of condensed tannins from *L*. *glaber* leaves in acetone-*d_6_*/D_2_O; *t*, terminal unit; *m*, middle unit; *e*, extension unit; PC, procyanidin; PD, prodelphinidin.

Figure 3 shows the ^13^C-NMR spectrum of condensed tannins from *L*. *glaber* leaves. The signal assignment was made based on the publication of Czochanska *et al*. [21]. The spectrum shows typical signals due to condensed tannins, containing PC and PD units. The signals at 116 ppm (C2´, C5´), 120 ppm (C6´), and 145 ppm (C3´, C4´) show the presence of PC units (catechin/epicatechin). The sharp and high signal at 146 ppm is typical of the presence of PD units (gallocatechin/epigallocatechin). The PC/PD ratio of condensed tannins is usually determined from the relative ratio of the peak areas at 145 ppm (C3´ and C4´ of PC) and 146 ppm (C3´ and C5´ of PD). In this case this relative ratio revealed that the condensed tannins are composed of 27.6% PC and 72.4% PD units. In addition, the predominant degradation products of (-)-epigallocatechin and 4β-(2-aminoethylthio) gallocatechin induced from the thiolysis degradation of condensed tannins showed a reasonable accordance with the ^13^C-NMR result. The region between 30 and 90 ppm is due to the signals of C2, C3, and C4 in ﬂavan-3-ol units. The two signals at 76 and 83 ppm were ascribed to 2,3-*cis* and 2,3-*trans* isomers, respectively. The spectrum demonstrated that both the stereoisomers co-exist, while the signal at 76 ppm was indicative of the majority presence of the *cis* form. The signals at 68 and 72 ppm were assignable to the C3 terminal and extension units, respectively.

### MALDI-TOF MS analysis

To obtain more detailed information on the chemical composition of condensed tannins, MALDI-TOF MS analysis was performed. Polymers with electronegative elements like oxygen or nitrogen are best cationized by Li^+^ or Na^+^[22]. We have examined various cationizing agents in the presence of DHB matrix for MALDI-TOF MS analysis of condensed tannins, but only Cs^+^ ions affected the intensity of the signals on the MALDI mass spectrum [23]. Using MALDI-TOF with deionization and selection of Cs^+^ as the cationization reagent rather than selection of Na^+^, condensed tannin polymers of higher DP were observed [24].

**Figure 4:**
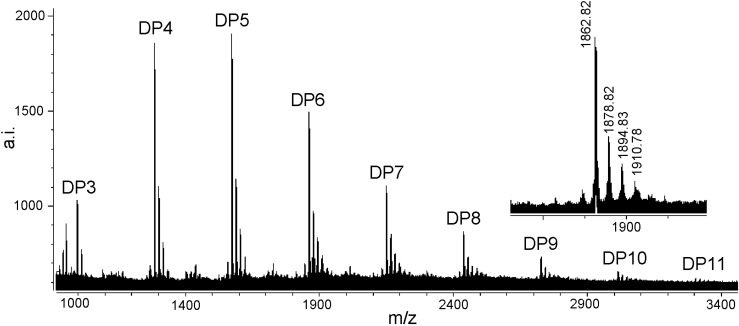
MALDI-TOF MS of condensed tannins from *L*. *glaber* leaves.

Figure 4 shows the MALDI-TOF mass spectrum of the condensed tannins isolated from *L*. *glaber* leaves. The masses of the highest peaks among the condensed tannin polymers with identical DP increased at the distance of 288 Da, corresponding to one catechin/epicatechin monomer [25]. The spectrum of the magnified hexamer (Figure 4) clearly indicated the mass increments of 16 Da, 32 Da and 48 Da. These masses indicated the presence of PD units, containing one more hydroxyl group at the aromatic ring B than PC units, although the sequence between PC and PD units could not be estimated. Given the absolute masses corresponding to each peak, it was further suggested that they contain procyanidin and prodelphinidin, as have already been indicated in the ^13^C-NMR spectrum. This result further indicated that the condensed tannins from *L*. *glaber* leaves did not contain galloylated sub-units like grape proanthocyanidins [26].

On the basis of the structures described by Krueger *et al*. [26], an equation was formulated to predict heteropolyflavan-3-ols of a higher DP (Table 2). The equation is 290 + 288*a* + 304*b* + 133, where 290 is the molecular weight of the terminal epicatechin unit, *a* is the degree of polymerization contributed by the epicatechin extending unit, *b* is the degree of polymerization contributed by the epigallocatechin extending unit, and 133 is the atomic weight of cesium. Application of this equation to the experimentally obtained data revealed the presence of a series of condensed tannins consisting of well-resolved polymers. The cesium adduct ion of condensed tannins [M + Cs]^+^ was detected up to undecamer and a degree of polymerization up to 7 was found in the positive-ion reﬂectron mode.

**Table 2:** Observed and calculated masses^a^ of heteropolyflavan-3-ols by MALDI-TOF MS.

Polymer	Number of catechin units	Number of gallocatechin units	Calculated [M + Cs]^+^	Observed [M + Cs]^+^
Trimer	3	0	999	998.81
	2	1	1015	1014.82
	1	2	1031	1030.88
Tetramer	4	0	1287	1286.76
	3	1	1303	1302.75
	2	2	1319	1318.78
	1	3	1335	1334.71
Pentamer	5	0	1575	1574.78
	4	1	1591	1590.84
	3	2	1607	1606.83
	2	3	1623	1622.85
Hexamer	6	0	1863	1862.82
	5	1	1879	1878.82
	4	2	1895	1894.83
	3	3	1911	1910.78
Heptamer	7	0	2151	2151.83
	6	1	2167	2167.78
	5	2	2183	2183.86
	4	3	2199	2198.87
Octamer	8	0	2439	2439.84
	7	1	2455	2455.86
	6	2	2471	2471.78
Nonamer	9	0	2727	2728.80
	8	1	2743	2743.85
	7	2	2759	2758.79
Decamer	10	0	3015	3015.83
	9	1	3031	3031.65
	8	2	3047	3048.18
Undecamer	11	0	3303	3305.33
	10	1	3319	3320.87
	9	2	3335	3336.78

^a^ Mass calculations were based on the equation 290 + 288*a* + 304*b* +133, where 290 is the molecular weight of the terminal epicatechin unit, *a* is the DP contributed by the epicatechin extending unit, *b* is the DP contributed by the epigallocatechin extending unit, and 133 is the atomic weight of cesium.

### Free radical scavenging activity

**Figure 5:**
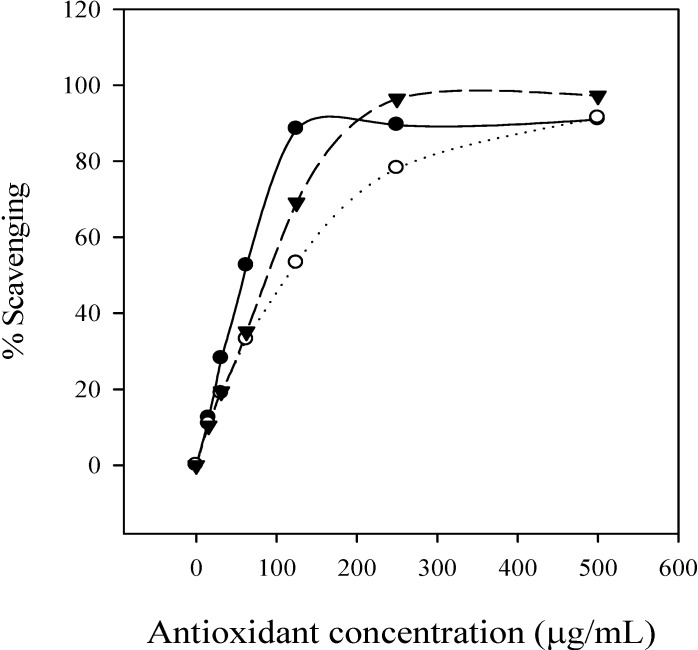
Percentage of free radical scavenging activity of condensed tannins, BHA and ascorbic acid. ●, condensed tannins of *L*. *glaber*; ○, BHA; ▼, ascorbic acid.

DPPH is a stable free radical and accepts electron or hydrogen radical to become a stable diamagnetic molecule [27]. In brief, the reduction capacity of DPPH was determined by the decrease in its absorbance at 517 nm, which is reduced by antioxidants [28]. The free radical scavenging activity increased with the increasing concentration of condensed tannins, ascorbic acid and BHA (Figure 5). By comparison of the corresponding IC_50_ values, the free radical scavenging activities, of the condensed tannins (59.51 μg/mL) and ascorbic acid (88.54 μg/mL) were higher than that of BHA (111.31 μg/mL), as indicated by the lowest IC_50_ value, showing that the condensed tannins from *L*. *glaber* leaves have a signiﬁcant free radical scavenging eﬀect.

## Conclusions

The thiolysis reaction products indicated the presence of the PC and PD structure. The relative ratio of the peak areas of 145/146 ppm in the ^13^C-NMR spectrum was reasonable for quantitative interpretation of the PC/PD ratio in the condensed tannins from *L*. *glaber* leaves. The result revealed that the condensed tannins contain PC (27.6%) and PD (72.4%), with the main constituent being the *cis* configuration of C2–C3. The MALDI-TOF MS results obviously proved the presence of PD units, and the maximum degree of DP was undecamer. The antioxidant properties of the condensed tannins were investigated through reduction of the DPPH free radical, and the obtained results showed that the polymeric fraction exhibited a potent free radical scavenging activity compared to BHA and ascorbic acid, suggesting that the condensed tannins from *L*. *glaber* leaves had the potent free radical scavenging activity.

## Experimental

### General

The solvents chloroform, acetone, methanol and hydrochloric acid were of analytical reagent (AR) purity grade. The CH_3_CN and trifluoroacetic acid (TFA) used for the analysis were of HPLC grade. *L*. *glaber* leaves were collected in Quanzhou, Fujian province, P.R. China. 1,1-Diphenyl-2-picrylhydrazyl (DPPH), cysteamine hydrochloride, ascorbic acid, butylated hydroxyanisole (BHA) and tannic acid were purchased from Aldrich (USA). (-)-Epicatechin (EC), (+)-catechin (Cat), (-)-epigallocatechin (EGC), (-)-gallocatechin (GC), (-)-gallocatechin gallate (GCG) and (-)-epicatechin gallate (ECG) were purchased from Sigma (USA). 4β-(2-Aminoethylthio) catechin (Cya-Cat), and 4β-(2-aminoethylthio) gallocatechin (Cya-GC) were prepared as described by Torres and Lozano [20] and identified by electrospray-mass spectrometry and nuclear magnetic resonance spectroscopy. Sephadex LH-20 was purchased from Amersham (USA). ^13^C-NMR spectra were recorded at 150 MHz in acetone-*d_6_*/D_2_O mixture with a Varian Mercury-600 spectrometer (USA).

### Extraction of condensed tannins, characterization and determination of total phenolics and condensed tannins

Leaf samples were taken to the laboratory immediately after collection and cleaned with distilled water. Fresh materials (120 g) were extracted thrice with 7:3 (v/v) acetone/water solution at 5°C. Condensed tannins were purified from *L*. *glaber* leaves, and the extraction and isolation procedures were described by Lin *et al*. [29]. The purified condensed tannins were characterized using acid-catalyzed degradation, ^13^C-NMR, and MALDI-TOF MS analysis. Total phenolics were measured with the Prussian Blue method [30], condensed tannins were assayed by the butanol-HCl method [31], using purified tannins from *L*. *glaber* leaves as the standards.

### Cysteamine degradation

A condensed tannin solution (4 mg/mL in methanol) was prepared. A sub-sample (50 μL) was placed in a vial and to this was added hydrochloric acid in methanol (3.3%, v/v; 50 μL) and cysteamine hydrochloride in methanol (50 mg/mL, 100 μL). The solution was heated at 40℃ for 30 min, and cooled to room temperature. The solution was ﬁltered (Φ13, 0.45 μm, Shenggong, China), and 10 μL of sample solution was analysed by HPLC. All incubations were repeated three times.

### Elution condition

The high performance liquid chromatograph was an Agilent 1100 system (USA) equipped with a diode array detector and a quaternary pump. A Hypersil ODS column (4.6 mm × 250 mm, 2.5 μm) (P.R. China) was used. Two solvents were used: A = 0.1% aqueous TFA; B = CH_3_CN. The elution system was: 0-5 min, 3% B (isocratic); 5-15 min, 3%-9% B (linear gradient); 15-45 min, 9%-16% B (linear gradient), 45-60 min, 16%-60% B (linear gradient). The column temperature was ambient and the flow-rate was set at 1 mL/min. Detection was at 280 nm and the UV spectra were acquired between 200-600 nm. Degradation products were identified on chromatograms according to their retention times and UV-visible spectra. Peaks were manually integrated, and quantification was performed by reporting the measured area into the calibration curve of the corresponding compound.

### MALDI-TOF MS analysis

The MALDI-TOF MS spectra were recorded on a Bruker Reflex III instrument (Germany). The irradiation source was a pulsed nitrogen laser with a wavelength of 337 nm, and the duration of the laser pulse was 3 ns. In the positive reflectron mode, an accelerating voltage of 20.0 kV and a reflectron voltage of 23.0 kV were used. The spectra of condensed tannins were obtained from a sum of 100-150 shots and calibrated using angiotensin II (1,046.5 MW), bombesin (1,619.8 MW), ACTHclip18-39 (2,465.2 MW), and somatostatin 28 (3,147.47 MW) as external standards. 2,5-Dihydroxybenzoic acid (DHB, 10 mg/mL aqueous solution) was used as the matrix. The sample solutions (7.5 mg/mL aqueous) were mixed with the matrix solution at a volumetric ratio of 1:3. The mixture (1 μL) was applied to the steel target. Amberlite IRP-64 cation-exchange resin (Sigma-Aldrich), equilibrated in deionized water, was used to deionize the analyte/matrix solution thrice. Cesium chloride (1 mg/mL) was mixed with the analyte/matrix solution at the 1:3 volumetric ratio to promote the formation of a single type of ion adduct ([M + Cs]^+^) [23].

### Free radical scavenging activity

The free radical scavenging activity was measured according to the method of Braca *et al*. [32]. A 100 µL of sample solution at different concentration (15-500 µg/mL) was added to 3 mL of DPPH solution (0.1 mM in methanolic solution). Thirty minutes later, the absorbance was measured at 517 nm. Lower absorbance of the reaction mixture indicates higher free radical scavenging activity. The IC_50_ value, defined as the amount of antioxidant necessary to decrease the initial DPPH concentration by 50%, was calculated from the results and used for comparison. The capability to scavenge the DPPH radical was calculated using the following equation: DPPH scavenging effect (%) = [(*A*_1_ – *A*_2_)/*A*_1_] × 100, where *A*_1_ is the absorbance of the control reaction and *A*_2_ is the absorbance in the presence of the sample. BHA and ascorbic acid were used as the controls.
